# Percutaneous Revascularization of Thrombotic and Calcified Coronary Lesions

**DOI:** 10.3390/jcm14030692

**Published:** 2025-01-22

**Authors:** Andrea Milzi, Federico Simonetto, Antonio Landi

**Affiliations:** 1 Division of Cardiology, Cardiocentro Ticino Institute, Ente Ospedaliero Cantonale, CH-6900 Lugano, Switzerland; andrea.milzi@eoc.ch; 2 Faculty of Biomedical Sciences, University of Italian Switzerland, CH-6900 Lugano, Switzerland; 3 Cardiovascular Department, Ospedale San Bassiano, 36061 Bassano del Grappa, Italy; federico.simonetto89@gmail.com

**Keywords:** calcified coronary artery lesions, thrombotic lesions, percutaneous coronary intervention, rotational atherectomy, orbital atherectomy, intravascular lithotripsy

## Abstract

Percutaneous coronary intervention (PCI) for thrombotic and heavily calcified coronary artery lesions and occlusions is often hampered by difficulty in wiring the occlusions, restoring antegrade flow, and proceeding to successful stent implantation. Characterization of dynamic anatomical features such as thrombi and the calcium distribution is key to prevent periprocedural complications and long-term adverse events, which are mainly driven by stent underexpansion and malapposition and may prompt in-stent restenosis or stent thrombosis. Therefore, multimodal imaging is a critical step during PCI to better characterize these high-risk lesions and select those in which careful preparation with debulking devices is needed or to guide stent optimization with the aim of improving procedural and long-term clinical outcomes. Hence, obtaining a better understanding of the underlying cause of thrombus formation, imaging the calcium distribution, and thorough planning remain crucial steps in selecting the optimal revascularization strategy for an individual patient. In this review, we summarize current evidence about the prevalence, predictors, and clinical outcomes of “hard-rock” thrombotic lesions treated by PCI, focusing on the value of imaging and physiological assessments performed to guide interventions. Furthermore, we provide an overview of cutting-edge technologies with the aim of facilitating the use of such devices according to specific procedural features.

## 1. Introduction

Lesions with large thrombus burden (LTB) account for up to 15–20% of patients with acute coronary syndrome (ACS) undergoing percutaneous coronary intervention (PCI), worsening procedural and clinical outcomes. Intravascular imaging is key for thrombus detection and quantification, and dedicated treatments (either pharmacological or interventional) may be used in select cases to reduce the risk of ischemic complications such as distal embolization, no reflow, and stent thrombosis.

Severely calcified coronary lesions are common among patients undergoing PCI, accounting for up to one-fourth of subjects presenting with ACS [1,2,3]. Treatment of these high-risk lesions still represents a clinical conundrum for interventional cardiologists due to the difficulty of wiring the occlusions and achieving optimal angiographic results following stent implantation. Large calcium deposits may hinder stent expansion and apposition, trigger damage to drug-eluting polymers, and hamper drug delivery and diffusion throughout the stent–vessel interface [4,5]. These effects could ultimately prompt periprocedural complications and long-term stent failure due to stent malapposition or underexpansion, with a consequent high risk of in-stent restenosis (ISR) or stent thrombosis (ST). Morphologic characterization of coronary artery calcification (CAC) by angiography alone is limited and needs to be complemented by intravascular imaging techniques such as intravascular ultrasound (IVUS) or optical coherence tomography (OCT) to allow optimal PCI planning [6,7,8].

Notably, extensive CAC and thrombi can coexist in patients with ACS, posing a “double threat” with unique challenges for an interventional cardiologist. Hence, a toolbox including intravascular imaging, advanced plaque modification modalities, and dedicated thrombus management devices is needed to achieve optimal PCI results. Within this framework, in this review we intend to summarize current evidence about the management of thrombotic and calcified lesions undergoing PCI, focusing on the value of imaging and physiological assessments performed to guide interventional treatments. Furthermore, we provide a brief overview of available and emergent devices, as well as a practical algorithm to guide the use of such technologies according to specific features of thrombotic and heavily calcified lesions.

## 2. Prevalence, Predictors, and Outcomes of Thrombotic and Calcified Coronary Lesions

Thrombus formation is part of the pathophysiological process leading to ACS. Lesions with LTB account for up to 60% of ACS patients [9] and are associated with a two- to four-fold increase in major cardiovascular events [10]. Thrombi usually arise from vessel wall damage, which may be represented by a ruptured or eroded plaque and/or a protruding calcified nodule. Thrombus formation is further accelerated by the enhanced prothrombotic milieu expressed by ACS patients. Occasionally, an intracoronary thrombus may be the result of the embolization of extracoronary material as a consequence of various conditions such as atrial fibrillation, dilated cardiomyopathies, endocarditis, intracardiac tumors, patent foramen ovale, systemic malignancies, or autoimmune diseases [11].

Primary PCI for calcified and complex coronary lesions has steadily increased over time due to the progressively aging population and the concomitant increase in comorbidities. Advanced age, arterial hypertension, chronic kidney disease, diabetes, and dyslipidemia are well-established risk factors associated with heavily calcified coronary lesions [2,3]. In a large, pooled analysis from the HORIZONS-AMI (Harmonizing Outcomes With Revascularization and Stents in Acute Myocardial Infarction) and ACUITY (Acute Catheterization and Urgent Intervention Triage Strategy) trials, moderate and severe target lesion calcifications were found in 26% and 6% of patients with ACS, respectively, including non-ST-segment elevation (NSTE) ACS and STEMI [12]. Although the pathophysiological mechanisms of STEMI are mainly related to the rupture of a soft, lipid-rich, friable plaque, there is mounting evidence showing that in 5–10% of patients the culprit lesion is an eroded calcified nodule [13]. Certain calcium morphologies, such as smaller calcifications (defined as “spotty” [14] or micro-calcifications [15]) and/or the presence of “pointy” superficial calcium deposits [16], are more frequent in ACS patients and may contribute to plaque instability [17]. Moderate and severe calcified lesions have been associated with greater risks of flow impairment, residual stenosis, dissection, and acute ST [12]. Moreover, a high calcium burden represents an independent predictor of poor long-term clinical outcomes, including major adverse cardiac events (MACEs), ST, and TLR at 1 year [12,18]. The prognostic impact of calcified lesions in ACS patients is apparently mitigated with newer-generation drug-eluting stents (DESs) [19]. Nevertheless, dealing with calcified lesions, especially in the context of ACS, carries a high risk of periprocedural and long-term complications related to several intertwined mechanisms. First, PCI for severely calcified coronary lesions is associated with an increased risk of stent malapposition and underexpansion (especially in the presence of a thrombus), which predisposes patients to ISR and ST [20,21,22]. Second, heavily calcified lesions may lead to structural injury to the polymer as a consequence of increased friction during stent delivery [23] or to limited drug delivery to the plaque through the calcification [24]. Finally, calcified lesions may—independent of the mechanic effects associated with PCI and stent implantation—trigger coagulation cascade activation and platelet aggregation. In particular, the severity of coronary calcification has been associated with the thrombin level [25] and high on-treatment platelet reactivity (HPR) in clopidogrel-treated patients undergoing PCI [26]. This prothrombotic status may further amplify the enhanced platelet reactivity in ACS patients, predisposing them—in conjunction with the above-mentioned stent-related mechanisms—to adverse outcomes.

Beyond the prognostic impact of CAC in ACS patients, it is important to highlight that CAC is also a marker of advanced atherosclerosis and correlates with the extension of the plaque burden [27]. Hence, CAC has been associated with both stent-related and -unrelated adverse cardiovascular events, such as all-cause and cardiovascular mortality and target lesion-related and -unrelated myocardial infarctions (MIs) treated with first- and second-generation DESs [1,28].

## 3. Intravascular Imaging and Physiology to Assess Thrombotic and Calcified Lesions

Despite good specificity, angiography has low accuracy in detecting CAC and quantifying its extension [29]. The rate of moderate-to-severe calcification rises from approximately 10% if the calcification extent is operator-reported [3,30] to 30–40% when angiograms are carefully evaluated by central core laboratories [12,28]. During a coronary angiogram, moderate calcifications are defined as radio-opaque deposits evident prior to contrast injection, while severe calcifications appear as linear areas that bilaterally follow the hypothetical silhouette of the coronary artery [31]. During contrast injection, it can be difficult to distinguish severe CAC from thrombotic material in ACS patients, as both may appear as “hazy” areas with contrast staining [32].

**Figure 1:**
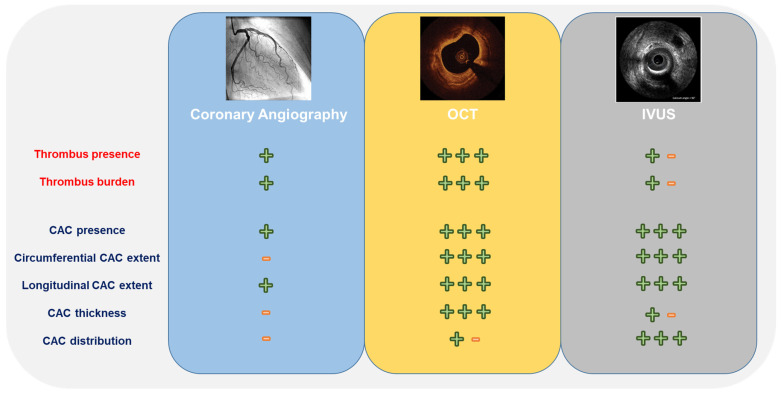
Characterization of thrombotic and heavily calcified coronary lesions by angiography and intracoronary imaging (intravascular ultrasound [IVUS] or optical coherence tomography [OCT]). Abbreviation: CAC, coronary artery calcification.

Conversely, intravascular imaging techniques such as IVUS and OCT enhance the differentiation of CAC from thrombi, allowing thorough planning and optimization of PCI procedures. Figure 1 shows the relative accuracy of angiography, IVUS, and OCT in the comprehensive assessment of CAC and its distinction from thrombi.

## 4. Intravascular Ultrasound (IVUS) and Near-Infrared Spectroscopy (NIRS)

A thrombus can be visualized using IVUS as an intraluminal mass with either an echo-lucent aspect or a variable gray-scale aspect with speckling or scintillation. The 2001 IVUS expert consensus document states that a diagnosis of thrombus using IVUS should be presumptive [33], taking into account the limited near-field resolution of this imaging method. Novel high-frequency IVUS systems have partially overcome this limitation, allowing a substantial increase in their ability to detect a thrombus and quantify its burden [34].

**Figure 2:**
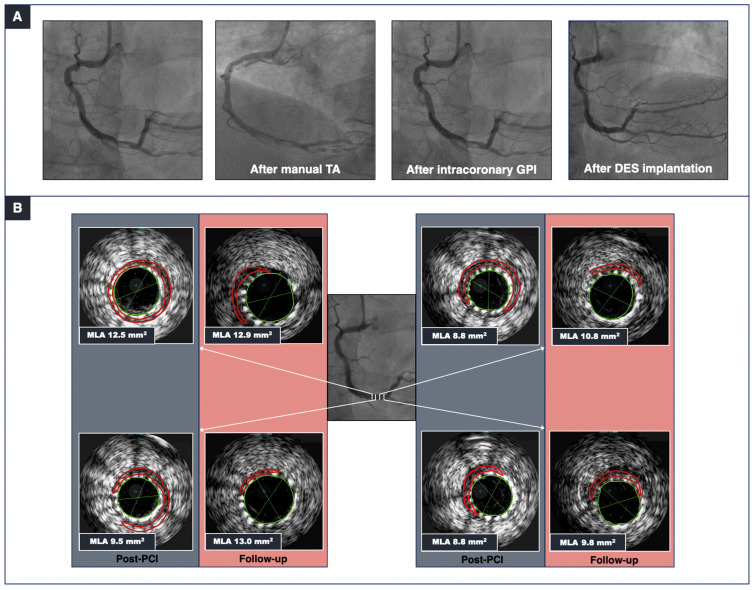
The mechanisms of stent expansion in a calcified thrombotic lesion. (**A**) Angiography revealed a thrombotic and calcified lesion in the right coronary artery of a patient presenting with non-ST-elevation myocardial infarction (left panel). After manual TA and intracoronary GPI administration (middle panels), a drug-eluting stent was implanted with a good angiographic result and no evidence of distal embolization. (**B**) IVUS (performed post-PCI and at a 5-day follow-up) documented a luminal gain 5 days after stent implantation. Abbreviations: DES = drug-eluting stent; TA = thrombus aspiration; GPI = glycoprotein IIb/IIIa inhibitor; MLA = minimal lumen area; IVUS = intravascular ultrasound; PCI = percutaneous coronary intervention.

IVUS enables the identification of calcified lesions as echo-lucent areas with posterior acoustic shadows. A typical characteristic of CAC is the presence of reverberations, concentric arcs reproduced at duplicated distances created by the oscillation of the ultrasound between the transducer and the calcium itself, which helps to differentiate CAC from fibrous tissue. IVUS allows the extents (in terms of the involved quadrants) and lengths of calcium arcs to be established (Figure 1). Due to the high penetration power of ultrasound, IVUS allows a more accurate assessment of calcium distribution and location compared with OCT. However, it is unable to fully appraise calcium thickness because the edge of the abluminal calcium is often hidden by the calcium shadow. Figure 2 shows IVUS images of calcific thrombotic lesions after PCI and at angiographic follow-up.

IVUS can be combined with near-infrared spectroscopy (NIRS) in the evaluation of plaque morphology. NIRS is based on the abilities of substances to absorb and scatter near-infrared light at different intensities, thus depicting the different components of atherosclerotic plaques [17]. Whereas NIRS can be employed to assess the extent of a necrotic lipid core, its clinical application in the evaluation of calcified and thrombotic lesions remains limited.

## 5. Optical Coherence Tomography (OCT)

OCT has the potential to overcome some limitations of IVUS, enabling accurate and complementary evaluations of thrombotic and calcified lesions.

OCT-based detection of thrombi is favored by the excellent resolution of this intravascular imaging modality. A thrombus appears as a compact intraluminal mass that can be attached to the luminal surface or free floating. OCT is capable of discriminating two different thrombus morphologies: red thrombi (with high backscattering and high attenuation due to the presence of red blood cells) and white platelet-rich thrombi, which are homogeneous with less backscattering [35].

**Figure 3:**
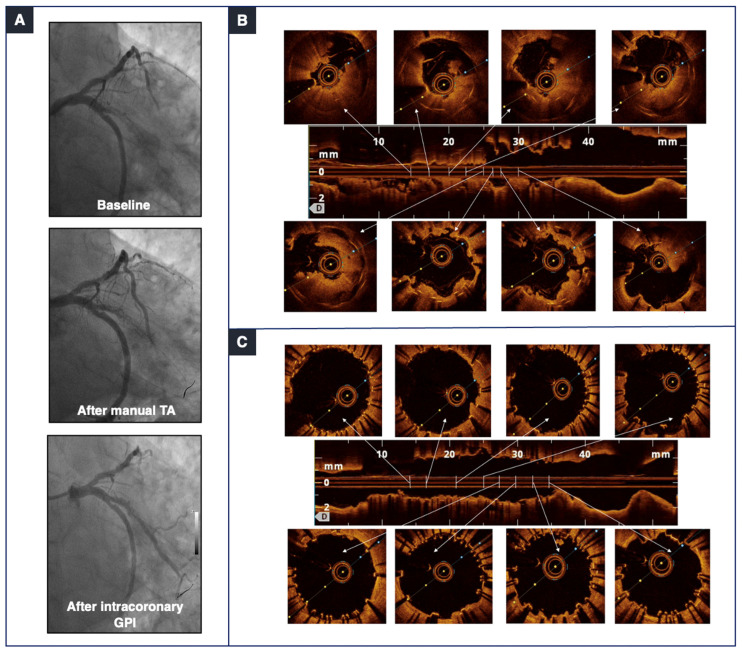
The value of optical coherence tomography (OCT) findings in thrombotic and calcified lesions. (**A**) During basal angiography (upper panel), we could appreciate an intrastent thrombosis on an obtuse marginal branch of a previously treated calcified lesion at baseline, after manual thrombus aspiration (TA) (middle panel), and after intracoronary glycoprotein IIb/IIIa (GPI) administration (lower panel). (**B**) A closer look by OCT confirmed a very large thrombus burden involving the stent lumen and the area around the struts. The final OCT (**C**) images show good apposition and expansion of the implanted drug-eluting stent, with minimal appreciable residual thrombus.

On OCT imaging, calcium appears as an area of attenuation or a heterogeneous zone with clearly delineated margins. OCT is more accurate than IVUS in the evaluation of the degree of the calcific arc and the calcium area, thickness, and length (Figure 1), which are recognized predictors of the response to pre-dilatation and stent expansion [36,37,38]. OCT has also a greater ability to detect the minimum luminal area, lesion length, reference diameters, and stent landing zones [39]. Figure 3 shows a case of late stent thrombosis of a previously treated calcified lesion. Here, insufficient lesion preparation resulted in stent underexpansion and malapposition, which ultimately led to this late complication.

## 6. Imaging Predictors of Resistant Lesions

In complex calcified lesions, intravascular imaging techniques provide significant upfront predictors of procedural success, which can be used to guide interventions.

Fujino et al. recently developed an OCT-derived calcium score to predict stent underexpansion (Table 1) in a cohort of patients with chronic coronary syndrome (CCS) treated without atherectomy or scoring devices [40]. This practical score included three OCT-derived parameters (a calcific arch > 180°, a calcific thickness > 0.5 mm, and a calcific longitudinal length > 5 mm) and was able to predict a significantly higher risk of stent underexpansion [41].

Similarly, Zhang et al. developed an IVUS-derived calcium score system in a mixed cohort where 30% were ACS patients [41] (Table 1). Using this score, a combination of at least two of four IVUS-derived morphological parameters (calcium > 270° for a length of at least 5 mm; 360° of calcium; a calcified nodule; and a vessel diameter < 3.5 mm) allowed good prediction of stent underexpansion. However, the systematic application of these scores (which have mostly been derived in CCS patients) to ACS patients, in whom calcium and thrombi frequently coexist, needs to be validated in further studies.

Intravascular imaging guidance is recommended by the European Society of Cardiology Guidelines for ACS patients undergoing PCI with a Class IIA recommendation [42]. The most recent CCS guidelines are even more supportive of the use of intravascular imaging to guide PCI for complex lesions (such as heavily calcified ones), assigning a Class I recommendation [43].

In addition to baseline high-risk features of CAC, intravascular imaging can also identify lesions that may benefit from calcium-modifying therapies that positively influence procedural and clinical outcomes. An elegant OCT study by Kubo et al. demonstrated that patients with calcium fractures had a significantly greater minimal stent area (5.02 ± 1.43 mm^2^ vs. 4.33 ± 1.22 mm^2^; *p* = 0.047) and stent expansion index (0.88 ± 0.17 vs. 0.78 ± 0.18; *p* = 0.030) compared to those without calcium fractures [44]. Of note, at angiographic follow-up, ISR (14% vs. 41%; *p* = 0.024) and ischemia-driven TLR (7% vs. 28%; *p* = 0.046) were significantly lower in patients with calcium fractures.

## 7. Coronary Physiology in Calcified and Thrombotic Lesions

Limited data exist about the value of functional assessment by fractional flow reserve (FFR) in calcified coronary lesions. From a theoretical standpoint, the impaired distensibility of an arterial wall could affect FFR values as the calcification extent increases. This hypothesis was proved in an observational study that enrolled 200 patients with intermediate lesions, which demonstrated that the correlation between angiographic severity and the FFR value decreases as CAC increases (no calcification or mild calcification: R^2^ = 0.24; moderate calcification: R^2^ = 0.11; severe calcification: R^2^ = 0.02) [45]. Additionally, a small observational study that enrolled 48 patients with NSTE-ACS demonstrated significant correlations between FFR values and the necrotic core volume (r = −0.497, *p* = 0.008) and the dense calcium volume (r = −0.332, *p* = 0.03) [46].

The use of the physiology of the culprit lesions of ACS patients prior to PCI is debated, considering that various factors such as the dynamic nature of coronary lesions in ACS (e.g., due to thrombus dissolution/apposition), the presence of microvascular dysfunction, and impaired vasodilation may limit the validity of FFR in this setting. A study by Leone et al. reported that using FFR as a “first intention” to determine the culprit lesions in NSTE-ACS patients is associated with higher rates of reinfarction and death compared with cases in which the culprit lesion is angiographically clear [47].

Coronary flow reserve (CFR) represents the ratio of the maximal hyperemic flow to the flow at rest; this reflects both the (fixed) obstruction due to the disease in epicardial vessels and the (dynamic) impairment of vasodilation of the microcirculation. In particular conditions, such as ACS with a large thrombus burden, CFR might be underestimated due to the maximal hyperemia at rest and/or severe acute microvascular dysfunction (e.g., due to distal microembolization). Furthermore, caution should be applied when interpreting CFR in heavily calcified lesions due to the potentially reduced distensibility of the vessel walls. In STEMI, poor microcirculation in the culprit vessel (depicted by a low CFR and/or increased microvascular resistance) is associated with an impaired prognosis and could be a target of future therapeutic interventions [48].

## 8. PCI for Complex Thrombotic and Calcified Coronary Lesions

Percutaneous revascularization for ACS patients with calcified, thrombotic lesions is hampered by the risk of distal embolization (DE) and no reflow and the need for adequate plaque/lesion modification to optimize stent implantation [49,50]. Among patients presenting with complex thrombotic and calcified coronary lesions, extensive lesion preparation is paramount to obtain optimal PCI results. Several dedicated tools are available to interventionalists in addition to the “traditional” toolbox including semi-compliant and non-compliant balloons. Dedicated consensus papers from European and North American societies have delineated various treatment algorithms for calcified coronary lesions [51,52].

However, the use of these devices is not free from specific challenges if thrombi and calcifications are concomitantly present.

Existing tools for calcium modification present some limitations in ACS patients, especially among those presenting with LTB. The presence of a thrombus is considered a relative contraindication for atherectomy due to the risk of no reflow caused by embolization of atheromatous debris and potential increased platelet activation caused by the rotating burr [53,54]. Furthermore, the safety of intravascular lithotripsy (IVL) in LTB lesions is unknown, as insonification of a thrombus by IVL may result in thrombus degradation/embolization. On the other hand, in severely calcified coronary arteries the deployment of dedicated tools for the management of lesions with LTB may be difficult, as they tend to be bulky and hard to deliver in complex anatomies.

Following successful lesion preparation, thrombotic and calcified lesions are usually treated by implanting drug-eluting stents (DESs). The role of drug-coated balloons (DCBs) in this setting deserves a careful discussion. A small study that enrolled 135 patients undergoing PCI for calcified de novo lesions showed comparable clinical outcomes with DCBs and DESs, with lower late lumen loss in the patients treated with DCBs [55]. Larger, prospective studies are needed to investigate the role of DCBs in this lesion setting.

Bifurcation PCI for thrombotic and calcified lesions also poses technical challenges. Since multiple rewirings and/or balloon crossings may be challenging in thrombotic and calcified bifurcations, a provisional stenting strategy (if feasible) without routine stenting of the side branch may be preferrable. This is in accordance with the principles expressed by the latest consensus paper from the European Bifurcation Club [56].

## 9. Thrombus Management in Heavily Calcified Lesions

### 9.1. Deferred Stenting

Deferring stenting in primary PCI has been investigated as an option to reduce DE and preserve microvasculature function, especially in LTB patients. Two randomized controlled trials (RCTs) investigated the value of deferring stenting with opposing results. The DANAMI 3-DEFER trial found no significant difference in the composite primary endpoint of all-cause mortality, nonfatal MI, or ischemia-driven revascularization for deferred vs. conventional stenting strategies in all patients undergoing primary PCI; in addition, routine deferred stenting was associated with higher rates of TVR [57]. Conversely, the DEFER-STEMI study randomized STEMI patients at high risk of no reflow to conventional and deferred stenting groups, showing a significant reduction in slow flow/no reflow in the deferred group (5.9% vs. 28.6%, *p* = 0.005) and a higher percentage of salvaged myocardium according to cardiac MRI [58]. These conflicting findings may be partly explained by the different patient selection protocols (patients at high risk of no reflow vs. an all-comer population) and the different uses of glycoprotein IIb/IIIa inhibitors (GPIs). Two subsequent meta-analyses found no significant difference in terms of MACEs between conventional PCI and deferred stenting [59,60].

Therefore, current guidelines recommend against deferred stenting as a routine strategy in STEMI patients [61]. However, since heavily calcified lesions frequently need additional strategies to obtain adequate plaque modification, deferred stenting may represent an alternative treatment strategy used to reduce the risks of extensive microvasculature damage, slow/no reflow, and DE.

### 9.2. Manual and Mechanical Thrombectomy

Different manual and mechanical thrombectomy devices have been developed to reduce the thrombus burden and the subsequent DE risk. Randomized clinical trials (RCTs) comparing routine thrombectomy and conventional PCI failed to demonstrate beneficial effects on hard clinical outcomes [62,63]; furthermore, thrombectomy was associated with an increased risk of stroke within the first 30 days in the TOTAL trial. However, in the subgroup of patients with LTB, defined by a TIMI Thrombus Score (TTS) ≥ 3, thrombus aspiration was associated with significant reductions in cardiovascular death and all-cause mortality, as well as an increased rate of cerebrovascular events at 30 days (0.9% vs. 0.5%, *p* = 0.04) in an individual patient-level meta-analysis [64]. Consequently, current guidelines do not support the routine use of such devices in STEMI patients [61]. However, thrombus aspiration and thrombectomy remain effective options for the treatment of select patients.

To date, no study has addressed the role of mechanical thrombectomy in the context of thrombotic and calcified lesions. The decision to employ mechanical thrombectomy in these patients has to be carefully evaluated on a case-by-case basis, taking into account potential advantages (such as a reduction in the thrombus burden, which may allow the subsequent use of advanced calcium-modifying techniques) as well as its specific challenges (among others, the difficult delivery of “bulky” thrombectomy systems with the potential for vessel damage and equipment entrapment).

### 9.3. Distal Protection Devices

Distal embolic filter devices include a capture wire and a catheter. The capture wire has a mesh filter bag at its distal tip, while the catheter is composed of a proximal recovery end and a distal delivery end. A standard guidewire is used to cross the lesion; subsequently, the catheter system is advanced over the guidewire and positioned in a predefined landing zone, usually 2.5–3 cm distal to the target lesion. At this point, the primary guidewire is retracted with the capture wire held in place; subsequently, the catheter is withdrawn, which allows exposure and deployment of the filter bag. Then, PCI is performed using the capture wire as the primary guidewire. Once the PCI is complete, the recovery end of the catheter is advanced over the capture wire for filter removal. The distal end of the recovery catheter is engaged with the proximal end of the filter and slowly advanced over it to allow for complete capture of the filter. The catheter and the capture wire are then cautiously removed together to ensure minimal dislodgment of debris material from the filter.

In comparison to other classes of embolic protection devices, distal devices maintain antegrade flow, thus minimizing the risk of ischemia and allowing optimal vessel visualization [65]. Potential drawbacks include the risk of DE due to the filter pore size and the size of the filter bag during the positioning/removal maneuvers. The pore sizes of the filter bags range from 100 to 110 µm. Therefore, the filter bags are only effective for capturing debris particles that are greater than these sizes. Another limitation of these devices is related to the large diameter of the delivery catheter, which predisposes patients to a higher risk of DE, complex maneuverability, and longer procedural times [65].

## 10. Calcium Modification in ACS Lesions with Large Thrombus Burden

### 10.1. Rotational Atherectomy

A rotational atherectomy (RA) (Boston Scientific, Marlborough, MA, USA) system is composed of a nickel-plated elliptic diamond-coated burr, available in sizes ranging from 1.25 to 2.5 mm in diameter, that works as an abrasive rotatory surface against calcific plaque [66]. The burr is mounted over an advancer (RotaLink) drive shaft connected to a motor that converts compressed gas into rotational energy [31]. The current system is controlled with an integrated RotaPro console, which allows single-handed use. The burr is advanced over a dedicated 0.009-inch wire (Rotawire). The wire is available in “floppy” and “extra-support” versions. Usually, to enhance deliverability, a regular 0.014-inch guidewire is used to cross the stenosis and is then exchanged through an over-the-wire balloon or microcatheter with the Rotawire. Recently, the Rotawire Drive wire was introduced. Due to its 1:1 torque, it is more steerable and can hence be used for direct wiring. The advised burr size/artery ratio is 0.5:0.6, and a burr revolution speed between 135,000 and 180,000 rpm is considered safe.

Potential complications of RA are slow/no reflow, burr lodging, coronary perforation, and transitory atrio-ventricular block [67]. The hypothesized mechanism for the slow/no-reflow phenomenon is the release of pulverized debris (5–10 μm) with subsequent distal propagation in the coronary vessel [50]. Slow/no reflow complicating RA has been reported to affect up to 24% of treated patients [68]. Factors associated with slow/no reflow can be divided into unmodifiable factors (such as the lesion length, moderate-to-severe angulation, and the reference vessel diameter) and modifiable factors (the initial burr-to-artery ratio and a short single run <15 s) [69]. Notably, the rotation speed of the burr has not been associated with the incidence of slow flow [68].

RA is relatively contraindicated in STEMI because of the potential increased risk of slow/no reflow due to embolization of thrombus debris [70]. The ROTATE trial analyzed 1308 patients undergoing RA for stable angina or ACS; RA had similar safety and angiographic outcomes in the two groups, and the rate of MACEs in ACS patients undergoing RA was comparable to that in a matched population of ACS patients not undergoing RA [71]. Kubler et al. found no differences in procedural success, periprocedural complications, or MACEs at 1 year among 207 consecutive patients undergoing RA for stable angina or ACS [70]. Additionally, in an observational registry study by Allali et al., angiographic success was high and similar between patients undergoing RA for ACS and stable angina. Furthermore, the rates of slow/no reflow were similar between the two groups (2.8% for stable angina vs. 0.8% for ACS, *p* = 0.32). In this registry study, the ACS patients were treated with a more aggressive antithrombotic regimen (GPI use: 9.2% vs. 1.8%, *p* < 0.001), and this can partly explain the difference in the slow/no-reflow rates between the groups [72]. Finally, a recent meta-analysis encompassing eight retrospective studies (n = 1237 ACS patients undergoing RA) showed reassuring safety data for RA, with a 7% incidence of periprocedural complications [73].

### 10.2. Orbital Atherectomy

An orbital atherectomy (OA) system consists of an eccentrically mounted diamond-coated 1.25 mm crown connected to a drive shaft and a controller powered by a pneumatic console (CSI Diamond 360, Coronary Orbital Atherectomy System, St. Paul, MN, USA). The crown is advanced over a 0.014-inch wire (available in only one version) with superior maneuverability compared to the 0.009-inch Rotawire [31]. The crown rotates with an elliptical orbit, which progressively increases in diameter as the rotation speed increases; this allows ablation of calcium with a 1.25 mm device in vessels up to 3.5 mm in diameter. The centrifugal force generated during rotation pushes and compresses the crown against the plaque with a “sanding” action on the calcified component [31]. The presence of diamond chips in the front and back of the crown allows plaque modification in both directions, making entrapment or lodging of the OA crown much less likely.

Evidence regarding the use of OA in ACS patients is limited. The ORBIT I and II trials showed high procedural success (the target lesion revascularization rate was 8.1% at 2 years in ORBIT II) and few periprocedural complications in severely calcified coronary lesions; however, patients with ACS or thrombotic lesions were excluded from both trials [74].

A multicenter retrospective registry study compared 454 patients undergoing OA for NSTE-ACS or CCS. It reported few complications, with no significant differences in procedural outcomes and no reflow between the two groups (no reflow: 2.0% in ACS vs. 0.5% in CCS) [75].

### 10.3. Intravascular Lithotripsy

An IVL balloon-catheter system includes miniaturized and arrayed lithotripters that are integrated into a semi-compliant balloon. The lithotripters generate shockwaves characterized by short durations (~5 mcs), which generate a peak positive pressure of ≈50 atm. The contrast-filled balloon is inflated at a sub-nominal pressure (4 atm) and apposed to the vessel wall, thus providing an effective fluid–tissue interface with similar acoustic impedances that facilitates efficient coupling of the shockwave energy and the vessel wall. Several mechanisms play a role in calcific plaque fragmentation by IVL, including axial splitting by compressive circumferential forces; the generation and violent collapse of cavitation bubbles inside the saline-contrast-filled balloon, which impact the surface of the calcific plaque; and progressive expansion of microfractures into macrofractures by the cumulative impact of multiple repetitive shockwave pulses.

The use of IVL in the setting of ACS has been poorly investigated. In the Disrupt CAD II and IV studies, only patients with CCS or unstable angina were enrolled, while in Disrupt CAD III, ACS was an exclusion criterion [76,77,78]. Potential complications related to the use of IVL in the setting of ACS include the need for prolonged balloon inflation with a risk of plaque/thrombus embolization and the R-on-T phenomenon, which may occur due to IVL-induced myocardial depolarization [79].

Cosgrove et al. performed a retrospective analysis of 72 patients that received IVL during primary PCI for STEMI [53]. IVL was mostly used for de novo culprit lesions (79%), followed by in-stent restenosis (15%) and underexpansion of a newly deployed stent (6%). The reported rate of procedural complications was low, and no reflow occurred in 4% of patients. In an analysis of the prospective BENELUX-IVL registry, which included 454 patients undergoing IVL, Oliveri et al. reported comparable procedural success rates and clinical outcomes between ACS and CCS patients [80].

### 10.4. Excimer Laser Atherectomy

Excimer laser coronary atherectomy (ELCA) induces plaque modification via photochemical, photothermal, and photomechanical actions. The current CVX 300 System (CVX-300 ELCA System, Spectranetics Inc., Colorado Springs, Colorado) uses a xenon chloride laser to generate energy with a laser catheter consisting of fiber arranged around a guidewire. Typical microparticles produced by ELCA are <10 μm in diameter and have a small impact on the microcirculation [81]. Catheters are available in four sizes ranging from 0.9 mm to 2.0 mm; these measurements correspond to the actual diameters of the intraplaque tunnels achieved with single passages of the laser catheters. The selection of the appropriate size is based on a catheter/vessel diameter ratio of 0.5:0.6 [31].

ELCA is a potentially useful strategy in patients with calcified lesions and LTB [82,83]. The multicenter CARMEL registry enrolled 151 ACS patients (65% with LTB in the culprit artery) [84]. The use of ELCA was associated with an improved TIMI flow grade, with the maximal effect observed in patients with LTB. To date, only one RCT has been performed in ACS patients. It demonstrated the safety and feasibility of ELCA use in this setting [85].

The clinical use of ELCA is limited. It is used as a bail-out strategy for lesions that are uncrossable and undilatable using other dedicated devices, with a reported procedural success rate of 93% [86]. ELCA can also be safely used in combination with RA via the so-called RASER technique [87].

### 10.5. Cutting and Scoring Balloons

Cutting balloons are non-compliant balloons with three or four microblades placed longitudinally on their surfaces. The microblades are intended to create incisions within an atherosclerotic plaque, allowing effective dilatation with lower inflation pressure [31]. Clinical trials failed to demonstrate the superiority of cutting balloons compared with conventional angioplasty [88].

Scoring balloons are low-profile semi-compliant balloons encircled by scoring elements; during inflation, the force is mainly exerted on the scoring elements, thus allowing focal concentration of the force and decreasing balloon slippage. Scoring balloons are more flexible and have a better profile compared with cutting balloons. Although not primarily intended for calcified lesions, the current main indication for the use of scoring balloons is the treatment of mild-to-moderate calcified lesions [89].

In the PREPARE-CALC trial, the use of cutting or scoring balloons was associated with similar stent expansion rates and similar clinical outcomes compared with rotational atherectomy [90]. The additional use of cutting balloons following rotational atherectomy did not improve stent expansion compared with the use of non-compliant balloons in the ROTA-CUT trial [91].

### 10.6. High-Pressure and Very High-Pressure Balloons

Non-compliant (NC) balloons can tolerate high inflation pressures with small increases in diameter, allowing more uniform force distribution along the vessel wall compared to compliant and semi-compliant balloons [92]. Repeated and prolonged inflations with NC balloons are considered the first choice for mild-to--moderate calcified lesions where the calcium arc is restricted (<90°).

A super-high-pressure balloon (OPN, Sys Medical, Fraunfeld, Switzerland) uses twin-layer technology to allow balloon inflation up to 35–40 atm with minimal increases in diameter [31]. A retrospective registry study showed successful use of OPN balloons in 326 patients with lesions that were undilatable using NC balloons, with high rates of procedural and angiographic success [93]. Other important indications for OPN use include the treatment of stent underexpansion and in-stent restenosis [94].

### 10.7. Phenotype-Based Calcium Management in ACS: Special Considerations

The most frequent calcification morphology present in the culprit lesions of ACS patients is represented by superficial calcium sheets, i.e., superficial calcium plates with intact fibrous caps and no protrusions into the lumen. These lesions are frequently associated with a higher incidence of white thrombi and higher stenosis severity. Superficial calcium plates may hinder stent expansion and apposition, especially if they are very extensive in terms of arc, depth, and length. For these lesions, all calcium-modifying techniques can be used successfully, keeping in mind that intravascular lithotripsy has the advantage of also fracturing deep calcium, whereas the effects of atherectomy devices usually remain limited to the more superficial layers.

Calcified nodules (CNs) are calcium masses with convex upper edges protruding into the lumen; these lesions represent a peculiar challenge due to suboptimal long-term results (as a consequence of CN protrusion) and a higher risk of mechanical complications. CNs represent up to 20–35% of coronary calcifications. In this subset of lesions, intravascular lithotripsy (with 1:1-sized balloons) and orbital atherectomy (usually with slow forward/backward device motion, a high speed >120,000 rpm, and a high number of passages) can be valuable options [51]; on the contrary, rotational atherectomy is very dependent on wire bias, and its use in the presence of CNs is associated with a 3-fold increase in long-term events [95]. A similar management strategy can also be advocated for calcified protrusions in which the calcium masses protruding into vessels are larger and without nodular aspects.

## 11. Conclusions

**Figure 4:**
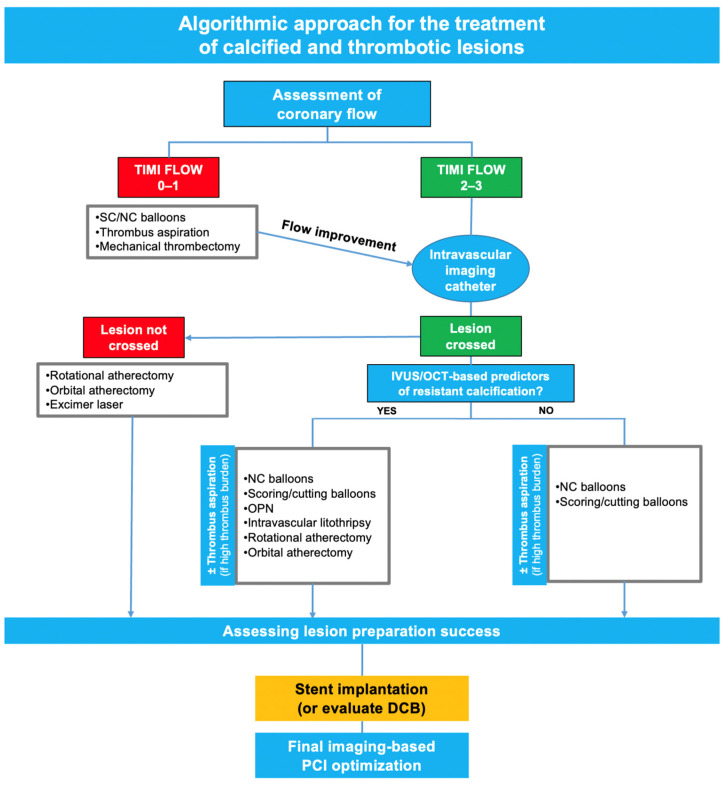
An algorithmic approach to the treatment of calcified and thrombotic lesions. Abbreviations: DCB: drug-coated balloon; NC: non-compliant; SC: semi-compliant; OPN: high-pressure balloons.

The simultaneous presence of thrombi and extensive calcification in patients with ACS still represents a significant challenge for interventional cardiologists, often leading to suboptimal PCI results. Integration of clinical and diagnostic information, careful lesion preparation guided by anatomical features (IVUS and OCT), and imaging-guided optimization of stent results are key for successful treatment in this high-risk setting. A potential algorithmic approach for the management of thrombotic and calcified lesions is depicted in Figure 4. A one-size-fits-all strategy does not apply to heavily calcified and thrombotic lesions, whose complex management requires thorough planning and a combination of tools and techniques to ensure optimal clinical outcomes.

## Figures and Tables

**Table 1:** Intravascular imaging-based calcium scores. Each score is computed by adding the points assigned to each parameter. Lesions with an OCT-based score of 4 and/or an IVUS-based score ≥ 2 present stent underexpansion more frequently, indicating a need for adequate lesion preparation. Abbreviations: IVUS, intravascular ultrasound; OCT, optical coherence tomography.

**OCT-based calcium score**		
**Maximal calcium angle (°)**	≤180°	0 points
	>180°	2 points
**Maximal calcium thickness (mm)**	≤0.5 mm	0 points
	>0.5 mm	1 point
**Calcium length (mm)**	≤5 mm	0 points
	>5 mm	1 point
**IVUS-based calcium score**		
**360° arc of calcium**	Absence	0 points
	Presence	1 point
**>270° arc of calcium with length > 5 mm**	Absence	0 points
	Presence	1 point
**Calcified nodule**	Absence	0 points
	Presence	1 point
**Vessel diameter (mm)**	≥3.5 mm	0 points
	<3.5 mm	1 point
